# Assessing microbial growth in drinking water using nucleic acid content and flow cytometry fingerprinting

**DOI:** 10.1016/j.isci.2024.111511

**Published:** 2024-12-01

**Authors:** Leila Claveau, Neil Hudson, Paul Jeffrey, Francis Hassard

**Affiliations:** 1 Cranfield University, College Road, Cranfield, Bedfordshire MK43 0AL, UK; 2 South East Water, Rocfort Road, Snodland, Kent ME6 5AH, UK

**Keywords:** Environmental science, Environmental health, Environmental chemistry

## Abstract

This study utilizes flow cytometry (FCM) to evaluate the high nucleic acid (HNA) and low nucleic acid (LNA) content of intact cells for monitoring bacterial dynamics in drinking water treatment and supply systems. Our findings indicate that chlorine and nutrients differently impact components of bacterial populations. HNA bacteria, characterized by high metabolic rates, quickly react to nutrient alterations, making them suitable indicators of growth under varying water treatment and supply conditions. Conversely, LNA bacteria adapt to environments with stable, slowly degradable organics, reflecting distinct physiological characteristics. Changes in water treatment and supply conditions, such as chlorine dosing and nutrient inputs, significantly impact the ratio between HNA and LNA. FCM fingerprinting combined with cluster analysis provides a more sensitive evaluation of water quality by capturing a broader range of microbial characteristics compared to using only HNA/LNA ratios. This work advocates for multi-parameter data analysis to advance monitoring techniques for water treatment and supply processes.

## Highlights

• Chlorine and nutrients impact HNA and LNA bacteria differently in drinking water
• HNA bacteria rapidly respond to nutrients, indicating faster growth potential
• Flow cytometry fingerprinting detects microbial shifts in water treatment and supply
• Combining flow cytometry and cluster analysis improves water quality monitoring

## Graphical abstract

**Figure:**
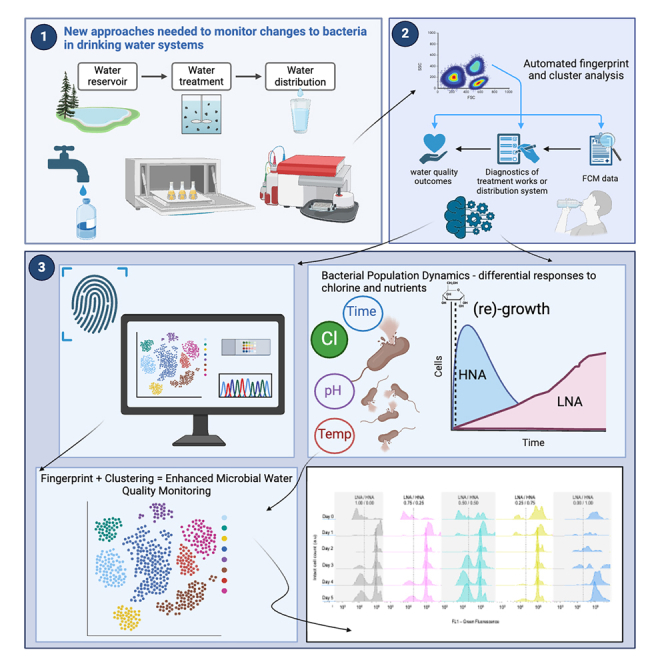

## Introduction

Assessing the bacterial status of drinking water is important for risk management, particularly as bacteria and pathogens in drinking water distribution systems (DWDSs) may withstand disinfection and under certain conditions multiply. The growing reliance on lower-quality water sources (such as reclaimed water) and the uncertainties introduced by climate change amplify these risks.^1^ This necessitates deeper understanding of bacterial community in water treatment and supply systems, where fluctuations in bacterial counts often signal inadequate water treatment or shifts in treated water quality,^2^ potentially compromising adherence to regulatory standards or creating conditions conducive for negative public health outcomes.^3^ Traditional culture-based methods for monitoring these bacteria are slow to undertake, are often strain-specific, and labor intensive as routine assessments in drinking water quality laboratories—despite being the gold standard approach.^4^ Building on previous studies, our work utilizes flow cytometry (FCM) in combination with SYBR Green I staining to rapidly and accurately fingerprint microbial populations in water treatment samples derived from an extended DWDS. This approach measures microbial abundance and provides added insights into cellular characteristics such as membrane permeability and cell size, providing an opportunity to monitor changes to the microbial populations through DWDS with greater precision.^5^^,^^6^ The nucleic acid content also serves as a surrogate for bacterial activity, as cells with greater nucleic acids content are either larger or undergrowing (re)growth in aquatic environments.^7^^,^^8^^,^^9^^,^^10^

Microbial populations are commonly identified through their designation as high nucleic acid content (HNA) and low nucleic acid content (LNA) bacteria, using FCM, exhibit varied physiological states and responses to their environments. HNA bacteria, larger and more metabolically active, are typically found in nutrient-rich conditions, while LNA bacteria, smaller and less active, tend to be more common in nutrient-poor conditions.^11^ Developing datasets on nucleic acid content-based classification of microorganisms may help elucidate how process factors such as chlorine disinfectants or assimilable nutrients affect these bacterial groups.^12^ The increased “bandwidth” FCM offers with which to measure responses to change drives insights into microbial populations within water treatment and supply processes^13^ as a broader range of microbial characteristics are captured than culture based assessment.^14^ Building on this hypothesis, a complementary approach is FCM fingerprinting of cell populations based on their fluorescence properties,^15^ which uses computation approaches to assess patterns and distributions of cells.^16^ Other techniques such as FCM coupled with amplicon sequencing, have expanded our insights into these populations by enabling characterization of cell abundance and diversity.^17^ However, the practical applicability of nucleic acid metrics for water quality assessment using FCM remains under discussion, as environmental factors such as pH and chlorine can alter fluorescence characteristics, thereby affecting how measurements should be interpreted, especially when distinguishing fluorescence background from cell events.^18^^,^^19^ Our study aims to address this challenge by exploring the interactions of free chlorine and nutrients with microbial populations, employing numerous FCM data analytics to monitor microbial dynamics at a water treatment works (WTWs) and its DWDS and through controlled growth experiments due to nutrient stimulation. This research fills a knowledge gap by providing a more comprehensive data analysis approach utilizing cluster analysis of fingerprints and machine learning to discriminate how bacterial populations change in drinking water. The work advances the understanding of microbial dynamics in DWDSs, contributing knowledge applicable to disciplines concerned with water quality.

## Results and discussion

### HNA and LNA dynamics in full scale DWDS

**Figure 1:**
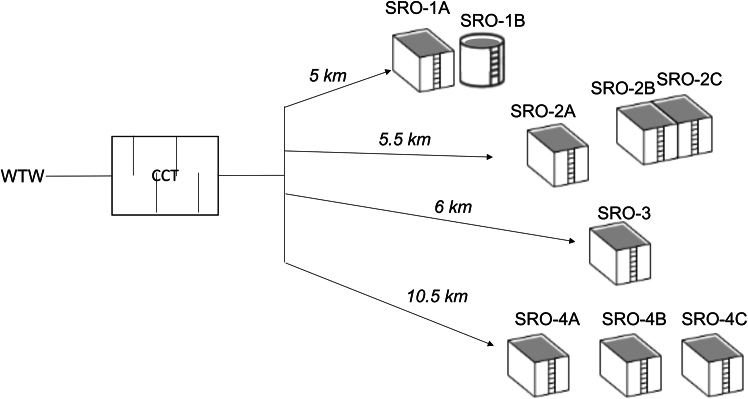
Schematic of the water distribution system, showing the path from a water treatment works (WTW) to various service reservoirs outlets (SROs) within the drinking water distribution systems (DWDS) Note not to scale and distances are approximate values based on water utility data.

**Figure 2:**
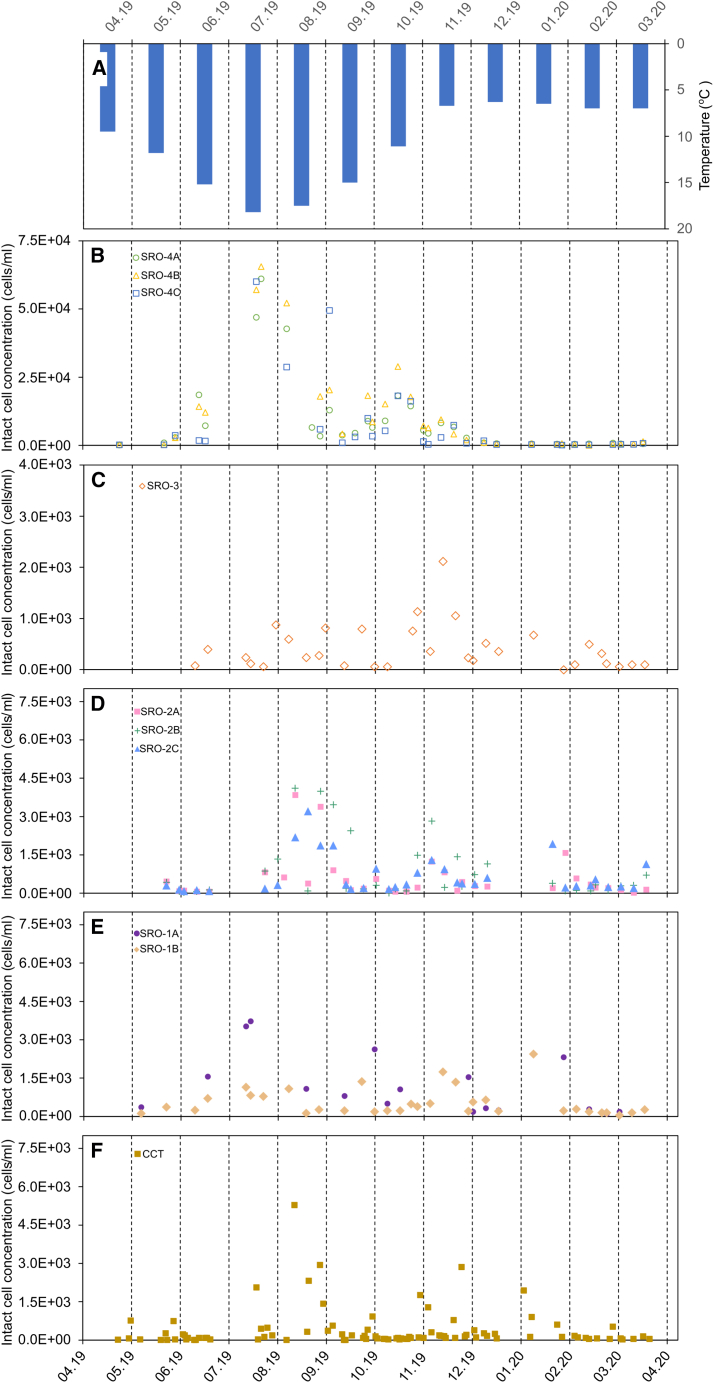
Intact cell count (ICC) data from chlorine contact tank (CCT) and service reservoir outlets (SRO) (A) The histogram represents the monthly average air temperature in the supply region. (B–E) are ICC for each outlet located at a distance of 5, 5.5, 6, and 10.5 km, respectively, from the CCT. (F) The graph depicts the CCT that feeds each of the SRs.

Cell concentration and viability were monitored weekly over a year across a chlorine contact tank (CCT) and nine service reservoir outlets (SROs), categorized into four groups by location and distance from CCT (Figure 1). Despite sourcing water from the same CCT, the average intact cell count (ICC) varied in the service reservoirs (SRs). For SR1 considering the two outlets had very different cell counts, with SRO-1A averaged 32,091 ICC/ml, while SRO-1B averaged 1,104 ICC/ml (*p* < 0.05, Mann-Whitney U test). The furthest SR (10 km) showed ICCs ranging from 7,168 to 11,692 ICC/ml, indicating potential microbial growth. Microbial growth within DWDS is influenced by increased water storage times and elevated temperatures. In this study, the temperature fluctuated between 6°C and 18°C (based on ambient air measures), which peaked in July 2019 (Figures 2B–2F). The SRO-2 (5.5 km) from the WTW had 891-1,086 ICC/ml (SRO-2A: 1,073 ICC/ml, SRO-2B: 891 ICC/ml, SRO-2C: 1,086 ICC/ml) that was similar to the SRO-3 (6 km) with an average of 1,087 ICC/ml (Figures 2C and 2D).

**Figure 3:**
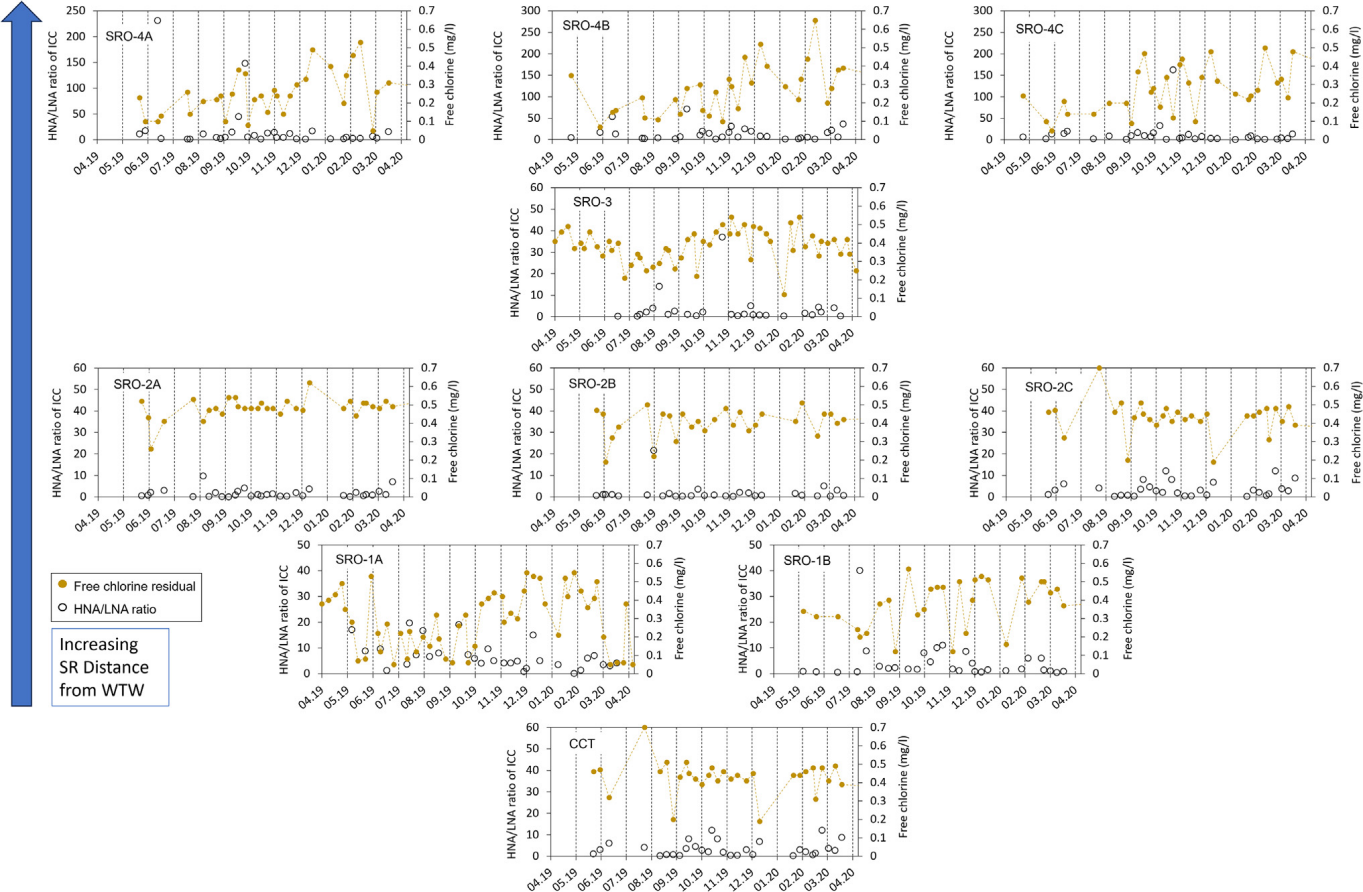
Ratio of high nucleic acid (HNA) and low nucleic acid (LNA) content (HNA/LNA) of intact cell counts (ICCs) and residual chlorine levels from the service reservoir (SR) measured at the SR outlets (SRO) and the chlorine contact tank (CCT) supplying them

Nucleic content in ICC the CCT and SRs was monitored over a year to evaluate the effects of treatment, conveyance, and storage on microbial sub-populations (Figure 3). The HNA/LNA was used to compare microbial composition within and across sites, where a ratio near 1 indicates a balance between HNA and LNA bacteria, ratios near 0 suggest predominance of LNA, and ratios above 1 indicate a higher count of HNA cells. The average HNA/LNA were as follows: SRO-1A at 5.01, SRO-1B at 4.26, SRO-2A at 3.29, SRO-2B at 4.62, SRO-2C at 3.29, SRO-3 at 5.36, and SRO-4A to -4C ranging from 12.07 to 18.42. The average HNA/LNA at the CCT was 4.33, signifying a larger proportion of viable HNA cells, compared to LNA. An average HNA/LNA of 4.33 at the CCT suggests a higher level of viable HNA cells compared to LNAs (relatively). The HNA/LNA, however, varied from 4.26 to 7.76 in the nearest SRs and 12.07 to 18.42 in the furthest, reflecting an increase in relative amounts of HNA cells compared to LNA cells with distribution system length. SRs 5.5 km from the WTW exhibited ratios fluctuating between 3.29 and 4.62, while those at 6 km averaged 5.36 (Figure 3). Chlorine levels were variable across the SRs, ranging from 0.05 to 0.65 mg/L, indicating inconsistency in residual chlorine concentrations within the DWDS a factor of chlorine decay and booster chlorination points (Figure 3). The variability in response to free chlorine influences fractions of the microbial populations differently, which would probably lead to a change in HNA/LNA.^19^ Ramseier et al.^2^ noted that HNA bacteria were more susceptible to oxidative damage from some disinfectants due to differences in cell membrane composition compared to LNA, although in this study, both types showed similar chlorine sensitivity. SRs located further from the CCT exhibited higher HNA/LNA, which could link to elevated water age, temperature variations, and their impact to chlorine decay—together producing conditions conducive for microbial growth.

The ICC variations at the SR located 5.5 km from the CCT (SRO-2s) showed trends consistent with those observed at the CCT outlet, with positive correlations (Spearman’s rank = 0.5–0.7, *p* < 0.05). Comparable patterns were seen in cell counts at SRO-1B and SRO-3, aligning with the CCT, except for SRO-1A that displayed no such correlation, suggesting aspects of this SR tank design and operations may influence bacterial water quality. However, cell counts at SRO-1A and the further SRO-4s, situated 10.5 km from the CCT, were statistically linked with temperature (Figure 3; Table 1). Random forest (RF) analysis showed that cell count variability at the CCT outlet significantly impacted the nearest SRs, with RF importance values of 0.658 for SRO-1B, 0.597 for SRO-2A, and 0.429 for SRO-2B. Temperature emerged as the main factor affecting cell counts at SRO-1A and the farther SRO-4s (RF importance = 0.450–0.547). This was not the case for SRO-3, were there was no relationship between cell count and temperature (RF importance = 0.515, *p* = 0.12). Strong negative correlations were found between free chlorine residuals and cell count variations across several SRs, underscoring chlorine’s role in inhibiting cell growth (Table 1). SRs farther from WTW with greater water storage times exhibit greater variability in cell counts due to temperature, chlorine decay and time for microbial growth to occur.^19^ Climate change is expected to negatively impact drinking water microbial quality, by raising DWDS temperature of DWDS and reducing raw water quality.^20^ Microbial populations are subject to multiple factors after the chlorine disinfection process at the WTW. Developing datasets on conditions of microbial community responses to environmental conditions will help generate better models of regrowth in DWDS and also guide disinfection strategies for better network management. The HNA/LNA is considered to be a useful metric that should be deployed alongside other measures of microbial water quality to assess water quality at WTW and DWDS. Our study provides insights into microbial dynamics across different SRs; however, the lack of network models on water storage time (so-called water age) limits our ability to fully interpret the impact of this variable.^21^ Future research is recommended incorporating detailed hydraulic assessments alongside advanced FCM data analytics to determine influence on microbial growth patterns and community shifts.

**Table 1:** Spearman’s rank correlation coefficient and random forest (RF) to identify relationships influencing variability in intact cell count (ICC) at the service reservoir (SR) outlet parameters include the ICC from chlorine contact tank (CCT), residual chlorine, temperature, high and low nucleic acid content ratio (HNA-LNA ratio)

Location	Test type	ICC from CCT	Residual chlorine	Temperature	HNA/LNA
SRO-1A	Spearman’s rank	0.24, *p* = 0.21	−0.32, *p* = 0.10	0.51, *p* < 0.05	0.22, *p* = 0.27
	Random forest	0.068	0.375	0.475	0.083
SRO-1B	SR	0.60, *p* < 0.05	−0.46, *p* < 0.05	0.32, *p* = 0.11	0.10, *p* = 0.61
	RF	0.658	0.045	0.109	0.188
SRO-2A	SR	0.48, *p* < 0.05	−0.21, *p* = 0.30	0.36, *p* = 0.064	−0.26, *p* = 0.19
	RF	0.597	0.131	0.077	0.194
SRO-2B	SR	0.51, *p* < 0.05	0.11, *p* = 0.59	0.023, *p* = 0.91	−0.36, *p* < 0.05
	RF	0.429	0.033	0.088	0.450
SRO-2C	SR	0.69, *p* < 0.05	−0.26, *p* = 0.20	0.096, *p* = 0.64	−0.41, *p* < 0.05
	RF	0.079	0.106	0.082	0.734
SRO-3	SR	0.59, *p* < 0.05	−0.58, *p* < 0.05	0.34, *p* = 0.12	0.28, *p* = 0.21
	RF	0.227	0.144	0.515	0.114
SRO-4A	SR	0.16, *p* = 0.26	−0.36, *p* < 0.05	0.71, *p* < 0.05	0.0074, *p* = 0.96
	RF	0.135	0.121	0.547	0.197
SRO-4B	SR	0.22, *p* = 0.10	−0.52, *p* < 0.05	0.78, *p* < 0.05	−0.042, *p* = 0.76
	RF	0.055	0.338	0.451	0.156
SRO-4C	SR	0.20, *p* = 0.15	−0.45, *p* < 0.05	0.65, *p* < 0.05	0.27, *p* = 0.055
	RF	0.062	0.204	0.450	0.285

### Assessing the impact of initial HNA/LNA on microbial regrowth potential

**Figure 4:**
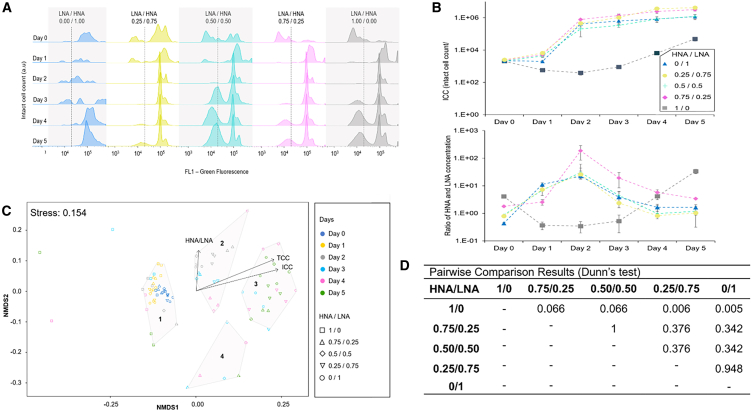
Daily intact cell growth and HNA/LNA ratios: experimental trends and multivariate analyses (A) Experimental measurements of the daily growth of intact cell count and their corresponding high and low nucleic acid content ratio (HNA/LNA) (B) ICC as a function of their initial HNA/LNA (1–0, 0.75–0.25, 0.5–0.5, 0.25–0.75, 0–1). Error bars show the error on the mean of repeat measurements. Results of Dunn’s Test for pairwise comparison of the resulting daily intact cell growth for different LNA/HNA. A single replicate’s fluorescence fingerprint is shown in (A), while cell concentration and HNA/LNA data represent mean values of repeat measurements, with error bars indicating ± propagated error term (A) and ±mean HNA/LNA error term in (B). (C) Non-metric multidimensional scaling (NMDS) plot of fluorescence fingerprints. (D) Results of pairwise comparisons for different HNA/LNA.

A five-day study was conducted, where the growth of native bacteria in water with varying HNA/LNA starting ratios (1/0 to 0/1) were monitored using FCM and fluorescent fingerprinting. Microcosms were deployed study the microbial growth in different populations of these bacteria derived the DWDS. Ratios of HNA/LNA were created and cell counts were normalized to ±20% via centrifugation and resuspended within dechlorinated water derived from the DWDS. Each sample was supplemented with 50 μg/L assimilable organic carbon (AOC), was incubated at 30°C in the dark, with daily assessments of nucleic acid content and fluorescence intensity (AU) of ICC. Initial ICC on day 0 was similar at 2,033 to 2,502 ICC/ml, with HNA/LNA across a gradient spanning 0.44 to 4.08. By day 5, cell counts had increased to 48,950–4.20 × 10^6^ ICC/ml, and HNA/LNA were from 1.03 to 33.46. The 1/0 HNA/LNA sample showed the slowest growth rate at 7,819 cells/day, experiencing an initial 80% drop in cell count by day 2, then a 123% increase by day 5. Fluorescence for this sample began to increase by day 4, from 34k to 437k AU. In contrast, samples with ratios from 0.75/0.25 to 0/1 exhibited higher growth rates ranging from 1.95 × 10^5^ to 5.26 × 10^5^ ICC/day (Figure 4). These samples peaked on day 2, then the ICC stabilized, displaying rapid initial growth, followed by the emergence of distinct bacterial communities by day 2. A pairwise Dunn’s test indicated that the 1/0 HNA/LNA ratio sample in the regrowth experiment was statistically distinct from other ratios (*p* < 0.066). There were no significant differences among the growth trends of the other ratios (*p* > 0.05).

Fingerprinting analysis of ICC confirmed that the HNA/LNA 1/0 sample maintained a unique profile throughout the five-day study, as evidenced by daily measurements clustering together, suggesting negligible change over time. Other HNA/LNA samples exhibited a clear growth pattern that converged together (Figure 4A). This stability was supported by silhouette, within-sum of squares (WSS), between-cluster sum of squares (BSS), and total sum of squares (TSS) values (0.55 and 0.42 for the unique sample, 0.44–0.47 and 0.06–0.14 for others, and 75%, respectively), which indicated strong internal cohesion and marked differences between unique versus other samples (Table 2). Similar growth patterns of cell subpopulations was observed and fingerprints clustered by day rather than sample type (exception HNA/LNA 1–0) (Figure 4C). Both fluorescent fingerprinting analysis and HNA/LNA showed convergence between samples toward the end of the incubation period on day 4, as all samples clustered together.

**Table 2:** Silhouette, WSS, and BSS/TSS values evaluating the cohesion of a sample within its cluster, the compactness of the cluster, and the separation of the clusters from each other respectfully

A	Cluster 1	Cluster 2	Cluster 3	Cluster 4
Silhouette	0.55	0.45	0.47	0.44
WSS	0.42	0.13	0.14	0.06
BSS/TSS	–	–	75%	–

B	Cluster 1	Cluster 2	Cluster 3	Cluster 4
Silhouette	0.54	0.38	0.49	0.45
WSS	2.23	2.10	0.81	1.83
BSS/TSS	–	–	80%	–

**Figure 5:**
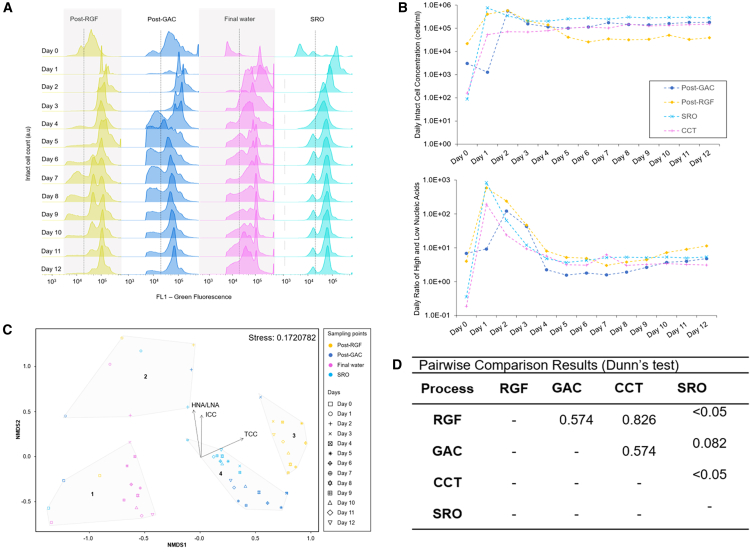
Experimental measurements of the daily cell growth of water coming from different stages of a water treatment works (WTWs) (A) The sample points post-rapid gravity filter (RGF), post-granular activated carbon (GAC), post-chlorine contact tank (CCT) and a service reservoir outlet (SRO) the daily fluorescence fingerprint of each sample. The intact cell count (ICC) variation on the y axis scaled for each sample to visualize the bacterial communities and their fluorescence. (B) The daily variation of the ICC (top) and high and low nucleic acid ratio (HNA/LNA) (lower). (C) NMDS plot obtained from a fingerprint analysis to evaluate the dissimilarities and similarities between and within the samples. The environmental vectors (ICC, HNA/LNA ratio) were included to determine the factors influencing the dissimilarities, 2D stress is indicated. (D) Pairwise comparison results to compare fingerprints between the different source waters after nutrient additional and regrowth experiment. A single replicate’s fluorescence fingerprint is shown in (A), cell concentration and HNA/LNA ratio data represent mean values of repeats, with error bars indicating mean concentration errors A and mean HNA/LNA errors in (B).

These values were calculated for the clustering undertaken in experiment A: Daily growth of intact cell count and their corresponding HNA/LNA (Figure 4C), and the experiment B: Daily cell growth of water coming from different stages of a WTW (Figure 5C).

The samples assembled into three distinct clusters, showing positive correlations with the non-metric multidimensional scaling (NMDS) axis 1 (NMDS1) and indicating moderate cohesion among samples. ICC vectors also positively correlated on this component, highlighting cell concentration as the differentiator in terms of fingerprint similarity. The HNA/LNA ratio had no correlation with the NMDS1 axis and showed a weak correlation with the NMDS2 axis. This suggests that the fluorescent fingerprints were more sensitive for measuring changes to the growing cell populations due to nutrient stimulation than HNA/LNA. A strong monotonic correlation (Spearman’s rank r = 0.9927, *p* < 0.001) between HNA/LNA and ICC in the unique sample, plateauing after day 4 as the HNA/LNA subpopulations became less distinct. The HNA/LNA and three bacterial communities identified in the fluorescence fingerprints. Community 1 showed the strongest correlation with the HNA/LNA (Spearman’s rank r = 0.96–0.51, *p* < 0.05), indicating its substantial influence on the ratio (RF importance ranges: 0.37–0.55), followed by community 3 (RF importance = 0.18–0.41), and community 2 (RF importance = 0.06–0.45). Thus community 1’s had a higher growth potential in response to and due to utilization of AOC substrate within the microcosm.

The responsiveness of some of the microbial communities but not others to AOC stimulation confirms that microbial populations in DWDS are dynamic, and probably species specific.^14^ Other examples of this phenomenon include, granular activated carbon (GAC) reactors that seed the water with HNA^22^ and slow sand filtration and membrane systems that can increase LNA through size selection mechanism^23^ contributing to changes in microbial community structure and function^11^ an aspect that should be explored further in DWDS. Alternative approaches such as single cell sequencing approaches are likely to drive further insights into growth of bacteria in aquatic systems.

### How do different cell populations regrow in DWDS?

In a 12-day study, variations in ICC and HNA/LNA were assessed in water derived from different WTW processes. Starting ICC readings varied widely, with GAC containing 3,012 ICC/ml, rapid gravity filter (RGF) 21,460 ICC/ml, while CCT and SRO were significantly lower at 159 and 88 ICC/ml, respectively, signaling a reduction in cell counts. The HNA/LNA was highest in GAC samples at 6.82, suggesting more HNA cells (relatively), while the lowest ratio found in CCT at 0.19 indicated a greater prevalence of LNA cells (relatively) post-chlorine treatment. From day 1, all samples except GAC showed a significant increase in ICC, with changes ranging dramatically from 1.51 × 10³ to 8.56 × 10^5^% (Figure 5B). Conversely, GAC experienced a 57.9% decrease in ICC but this trend reversed by day 2, as ICC and HNA/LNA increased. By day 4, HNA/LNA across all samples had stabilized suggesting that microbial responses to a single pulse of AOC were primarily HNA. The cell count equilibrates to a steady state and higher level than at the start of the experiment. The HNA/LNA appeared to rapidly increase in days 1–3 before declining back to near baseline in days 4 onwards. This provides some circumstantial evidence of flexibility in population level responses. Further work should investigate if HNA/LNA change is a species level response or rather phenotypic change in response to nutrient availability.

**Figure 6:**
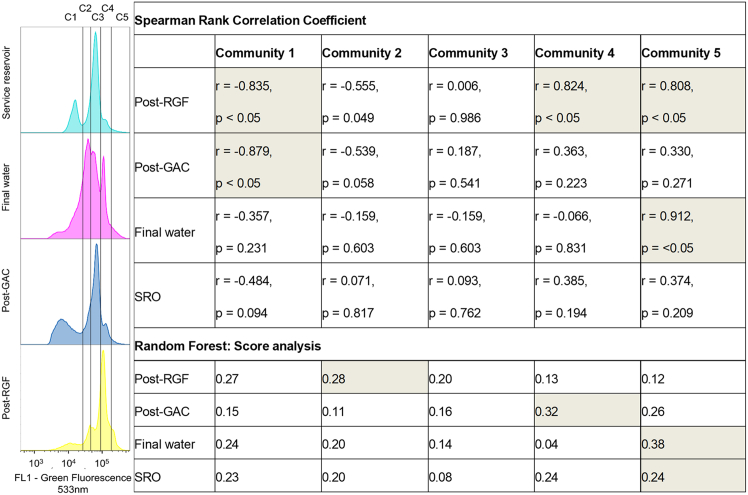
Fingerprint of each water at the end of the regrowth experiment (A) The clusters C1–C5 represent communities of cells. (B) Spearman’s rank correlation coefficient and random forest analysis to determine and evaluate the monotonic relationships between the daily variation of the high and low nucleic acid ratio (HNA/LNA) cell population and the daily variation of each community composing the HNA and LNA subpopulations of each treatment processes.

Fluorescence fingerprinting revealed that treatment-specific microbial profiles were largely retained during the experiment, with limited overlap between treatment types (Figures 5C and 5D). Supported by silhouette, WSS and BSS/TSS values (0.38–0.54 and 0.81–2.23, and 80%, respectively), these findings highlighted distinct microbial communities among the samples. Daily monitoring of each sample’s fluorescent fingerprint identified five distinct microbial communities, labeled C1 through C5. These ranged in fluorescence intensities from 2.2k AU for C1 to 612k AU for C5 (Figure 6A). The RF analysis showed the primary influences on the HNA/LNA, identifying different drivers for each water type: RGF influenced primarily by communities 1 and 2; GAC by community 4; and final water by community 5, with SRO influenced by communities 1, 4, and 5 (Figure 6B). These findings demonstrate how microbial communities change in water treatment systems. The rapid response of HNA populations to nutrient stimulation provides a basis for use of the HNA/LNA as a biomarker within DWDS. We acknowledge the benefits of capturing a broader range of microbial characteristics and the HNA/LNA ratios represent an oversimplification of complex microbial phenomena. We advocate for the use of advanced monitoring techniques like FCM fingerprinting for better monitoring within aquatic systems^3^^,^^15^^,^^24^ as they are highly amenable to automation through machine learning approaches.^25^

### Future perspectives on FCM as a monitoring technique

FCM provides rapid, reproducible measurements of bacteria and their nucleic acid content, effectively distinguishing between sub-populations, complementing traditional culture-based methods. This capability is useful for identifying active bacteria that are resistant to disinfection and prone to regrow under varying environmental conditions such as changes in nutrient levels, temperature, or disinfectant residuals. Throughout the year-long monitoring at the CCT and several SROs, FCM detected significant variability in microbial populations influenced by environmental factors like temperature and probably the water age/storage time. This suggests that the HNA/LNA could serve as a quick and reliable indicator of bacterial growth, particularly HNA subpopulations during extended water storage. FCM proved valuable in rapidly identifying distinct microbial communities across different treatment types and tracking their temporal shifts.

Integrating FCM and especially fluorescent fingerprinting into routine water quality monitoring practices is recommended to enhance microbial management in DWDS. This integration would facilitate a more proactive approach to water safety management due to the sensitivity in which it can detect changes to microbial populations and thus help maintain compliance with health regulations. Insights from FCM can inform the development of alternative water treatment and distribution strategies that adapt to changes in microbial populations.

The study also opens avenues for further research into applying FCM combined with advanced analytical methods like fluorescence fingerprinting and cluster analysis. These methods could be further developed to automate FCM analysis using artificial intelligence and machine learning, refining our ability to predict and control the microbial quality of drinking water. Such advancements could improve public health protection and system efficiency by enabling rapid, data-driven decisions. By advancing traditional microbial monitoring, FCM offers a promising future in water quality management, setting a standard for effective monitoring of microbial populations in drinking water systems.

### Limitations of the study

This research was conducted on a single WTW and its DWDS in Southeast England, which may limit the applicability of the findings to other regions with different water sources or infrastructures. The absence of detailed hydraulic data, such as precise water age and flow patterns, constrains the understanding of how residence time affects microbial growth and community composition. While FCM fingerprinting enhances insight into microbial populations, it does not provide species-level identification, somewhat limiting the ability to pinpoint specific bacterial taxa. Environmental factors like pH and additional nutrient sources were not extensively monitored, potentially influencing bacterial activity beyond the measured variables. Lastly, laboratory regrowth experiments, while carefully controlled, may not fully capture the complexities of real-world distribution systems, including biofilm interactions and variable operational conditions expected at full scale.

## Resource availability

### Lead contact

Further information and requests for resources and reagents should be directed to and will be fulfilled by the lead contact, Dr Francis Hassard (francis.hassard@cranfield.ac.uk).

### Materials availability

This study did not generate new unique reagents.

### Data and code availability

• Data supporting this study are openly available from figshare at (https://doi.org/10.6084/m9.figshare.27902202) and are publicly available as of the date of publication.
• This study does not include original code.
• Any additional information required to reanalyze the data reported in this paper is available from the lead contact upon request

## Acknowledgments

The UK Engineering and Physical Sciences Research Council (EPSRC) and South East Water funded the work through an Engineering Doctoral Training Award (grant number: EP/L015412/1) to L.C.. The authors gratefully acknowledge South East Water for providing access to drinking water treatment works and support with sampling and water analysis.

## Author contributions

Conceptualization, F.H. and L.C.; methodology, L.C. and N.H.; investigation, L.C.; writing – original draft, L.C; writing – review & editing, P.J., F.H., and N.H.; funding acquisition, F.H.; supervision, F.H. and P.J.

## Declaration of interests

The authors declare no competing interests.

## STAR★Methods

### Key resources table

**Table:** 

REAGENT or RESOURCE	SOURCE	IDENTIFIER
**Chemicals, peptides, and recombinant proteins**		

SYBR Green I Nucleic Acid Gel Stain (10,000x Concentrate in DMSO)	Thermo Fisher Scientific	Cat# S7563, CAS# 163795-75-3
Propidium Iodide (PI)	Sigma-Aldrich	Cat# P4170, CAS# 25535-16-4
Sodium Thiosulfate Solution (10% w/v)	Merck	Cat# 106513, CAS# 7772-98-7
Sodium Acetate Anhydrous (ACS Grade)	Sigma-Aldrich	Cat# S2889, CAS# 127-09-3
Milli-Q Water (purified using Direct-Q® 3 UV System)	Millipore	Model# ZRQSVP3WW, CAS# N/A

**Deposited data**		

processed data have been deposited at Figshare and are publicly available as of the date of publication. Raw data are available on request.	Figshare	(https://doi.org/10.6084/m9.figshare.27902202)

**Software and algorithms**		

Flow Cytometry Analysis Software (FlowJo v10)	Becton, Dickinson and Company	RRID: SCR_008520
Cytometric Histogram Image Comparison (CHIC) method,	Koch et al.^26^	https://doi.org/10.1002/cyto.a.22298

### Method details

#### Monitoring full-scale water treatment works and its drinking water distribution systems

A WTW and its associated DWDS, including four SR, were monitored weekly over one year. The study aimed to assess ICC, viability, and nucleic content at various points in WTW and DWDS. The WTW, located in Sussex, Southeast England, sources water from a river and a borehole, with the blend ratio influenced by water quality and abstraction permits. It features an aerator, a clarifier, microfiltration membranes, UV, and chlorine disinfection via a CCT. The SRs, situated 5 to 10.5 km from the WTW, operate independently with only supply from this feed. They vary in design and age: SRO-1A and B,approximately 100 years old, serve 6,686 people, with SRO-1B being circular. SRO-2A and SRO-2B, both 89 years old, and SRO-2C, 54 years old, collectively serve 6,553 people. SRO-3, 68 years old, supplies water to 15,123 individuals, and the 47-year-old SRO-4A, B, and C cater to 11,767 people. All reservoirs are made of reinforced concrete, except for SRO-2B, which is composed of stainless steel (Figure 1).

#### Simulated regrowth of different HNA-LNA ratios

To evaluate bacterial populations, two bench-scale experiments utilized 2-L samples from two different SRs in the South England. These samples, identified for their stable nucleic acid profiles determined over a 24-month period, which contained approximately 80% HNA content and 75% LNA content, respectively, representing the highest proportions of each within natural microbiota from earlier work.^15^ Samples were collected by flushing taps for 3 min, flame sterilizing for 30 s, and then flushing again for 30 s before collection in sterile 250 mL bottles with sodium thiosulfate to neutralize residual chlorine. Samples were transported to the lab within 8 h, maintained at 4°C–8°C and experiments commenced within 24 h. A subsample of LNA water was filtered (0.22 μm and 0.10 μm) to achieve a cell-free matrix verified by FCM with cell counts below the limit of detection (LOD).^27^ We prepared mixtures from the samples in various HNA-LNA ratios (ranging from mostly HNA to mostly LNA: 1–0, 0.75–0.25, 0.50–0.50, 0.25–0.75, 0–1) in 250 mL AOC-free bottles. These were uniformly spiked with 100 μg of sodium acetate as a carbon source and incubated at 30°C. Over six days, we monitored cell growth and the relative abundance of HNA and LNA.

To assess the consistency of regrowth effects across different SR and DWDS, five 1-L samples with varied HNA-LNA ratios (0.1–0.8 HNA) were collected from distinct sites in South England. These sites were selected to include a GAC filter (0.7–0.3 HNA/LNA), slow sand filter outlet (0.6–0.4 HNA/LNA), a CCT (0.1–0.9 HNA/LNA), and a service reservoir outlet (SRO – 0.8–0.2 HNA/LNA), chosen for their diverse HNA/LNA, which differ from those in earlier experiments. After collection, the samples were processed to prepare cell-free water and enhanced with AOC as before. Over 13 days, the regrowth experiments tracked daily variations in cell growth and HNA and LNA levels. Small volumes (<1 mL) were carefully extracted using sterile plastic syringes to minimize disturbances to the cell populations.

#### Physico-chemical and flow cytometric measures

Physical parameters included the monthly average air temperature in Sussex, sourced from the Met Office (UK). Measurement of chemical parameters, such as residual chlorine, followed regulatory guidelines.^28^ Duplicate samples were tested for turbidity, pH, and both total and free chlorine residuals using the Standard Methods for the Examination of Water and Wastewater.^29^ Turbidity was measured using the nephelometric method (2130 B), pH by method 4500, and chlorine residuals by the DPD colorimetric method (4500-Cl G). FCM measurements for total cell counts and intact cell counts alongside secondary gating for HNA and LNA bacteria were determined using common approaches.^15^^,^^27^ FCM was conducted using dual-stain fingerprinting with SYBR Green I and propidium iodide (PI). FCM analysis was performed using a dual-staining method with SYBR Green I and PI. SYBR Green I stains nucleic acids in all cells, while PI selectively penetrates cells with compromised membranes. This allows for the identification of intact cells (SYBR⁺/PI⁻) and compromised cells (SYBR⁺/PI⁺), providing insights into the microbial community’s physiological status. Additionally, studies have shown a strong correlation between intact cell counts obtained via FCM and microbial activity in aquatic systems.^6^ For FCM fingerprinting the intact cell counts distribution was used (instead of total cell counts) as it better reflects the biodiversity changes to microbial populations in treatment and supply systems where chlorine as a disinfectant.^30^

### Quantification and statistical analysis

#### Flow cytometry data analysis

Cell concentration, ICC, TCC, HNA, and LNA data were extracted from FCS files using FlowJo software (v10.7.2), which facilitated FL1 vs. FL3 dot plot extraction. Image comparison was conducted using ImageJ (v1.54days) employing the Cytometric Histogram Image Comparison (CHIC) method. This followed guidelines adapted from Koch et al. (2013), using the Bray-Curtis Dissimilarity index to compare images based on pixel (cell) density and distribution. Results visualization and significance assessment were performed with the R statistics engine (2022−02−3), employing NMDS and vector analysis, correlating predictor variables with CHIC fingerprints using Pearson’s rank correlation. 2D stress was calculated as a measure of how well the NMDS configuration reproduces the observed dissimilarities or distances among the data points in a two-dimensional space. Stress values < 0.05: Excellent fit; 0.05–0.1: Good; 0.1–0.2: Acceptable with caution; 0.2–0.3: Poor fit. In our NMDS analysis, cluster validation involved the Silhouette Value, Within-Cluster Sum of Squares (WSS), and the ratio of Between-Cluster Sum of Squares to TSS (BSS/TSS, presented in Table 2). A Silhouette Value near +1 indicates strong cluster cohesion and separation, assessing clustering quality and optimal cluster count. WSS measures cluster compactness; lower values signify tighter, more cohesive clusters. The BSS/TSS ratio evaluates how well the clustering partitions the data into distinct groups, with values close to 1 indicating well-separated clusters. We identified the optimal cluster number using the elbow method on WSS, determining where additional clusters did not significantly decrease WSS.

#### Additional data analysis

Statistical relationships between HNA and LNA abundance and other variables were examined using Spearman’s rank correlation coefficient and an RF algorithm (Table 1) to identify predictors of bacterial growth patterns. Dunn’s test and the Mann Whitney U test (Figure 4) provided detailed comparative analysis of the datasets, helping determine statistical significance between experimental groups and variables.
